# Selection of the Best Model of Distribution of Measurement Points in Contact Coordinate Measurements of Free-Form Surfaces of Products

**DOI:** 10.3390/s19245346

**Published:** 2019-12-04

**Authors:** Marek Magdziak

**Affiliations:** The Faculty of Mechanical Engineering and Aeronautics, Rzeszów University of Technology, al. Powstańców Warszawy 12, 35-959 Rzeszów, Poland; marekm@prz.edu.pl; Tel.: +48-178-651-491

**Keywords:** coordinate measuring technique, measurement strategy, free-form surface, AHP, measurement accuracy

## Abstract

The article presents a new method for determining the distribution of measurement points, which can be used in the case of contact coordinate measurements of curvilinear surfaces of products. The developed method is based on multi-criteria decision analysis. In the case of the new method, the selection of the distribution of measurement points on free-form surfaces is carried out based on the analysis of five different criteria. The selection of the best model of the distribution of measurement points results from the accuracy of coordinate measurements, the time needed to complete measurement tasks, the number of measurement points, the accuracy of the substitute surface representing the measured free-form surface and the area where measurement points are located. The purpose of using the developed method of the distribution of measurement points is to increase the performance of coordinate measurements primarily by increasing the automation of strategy planning of measurements of curvilinear surfaces and improving the accuracy of measurements of free-form surfaces of products. The new method takes into account various aspects of coordinate measurements to determine the final model of the distribution of measurement points on measured surfaces of products, thereby increasing the probability of the proper determination (i.e., identifying the highest deviations of a product) of the location of measurement points on the surfaces of a measured object. The paper presents an example of the application of the created method, which concerns the selection of the best model of the distribution of measurement points on a selected free-form surface. This example is based on, among others, the results of experimental investigations, which were carried out by using the ACCURA II coordinate measuring machine equipped with the VAST XXT measuring probe and the Calypso measurement software. The results of investigations indicate a significant reduction in time of coordinate measurements of products when using the new method for determining the distribution of measurement points. However, shortening the time of coordinate measurements does not reduce their accuracy.

## 1. Introduction

The coordinate measuring technique is widely applied under industrial conditions. Various coordinate measuring systems are presently used by many measurement laboratories and different companies representing various branches of the industry. A coordinate measuring machine (CMM) is still a very popular measuring system. CMMs may work in two main modes (i.e., the contact and non-contact modes), which may be applied because of using different types of measuring probes [1,2]. The user of a CMM must take into account a lot of factors to properly create a measurement program, which controls the work of a coordinate measuring machine. One of the factors, which has a significant influence on the accuracy of contact coordinate measurements, is the measurement strategy, planning of which takes place at the stage of preparation of measurement programs controlling the work of coordinate measuring machines. Other factors that affect the accuracy of coordinate measurements are, among others, environmental conditions prevailing in a measurement laboratory, the accuracy of applied measuring probes and the level of training of persons performing coordinate measurements. The detailed review of the factors determining the final results of coordinate measurements and measurement uncertainty is presented in the work [3].

In the coordinate measuring technique different measurement strategies may be applied at different stages of coordinate measurements of products. Therefore, the strategy of contact coordinate measurements may include a lot of elements, e.g., applied algorithms of the probe radius correction process [4], scanning velocity [3], the methods of data processing [5], a number of measurement points and their distribution on measured surfaces of analyzed products [6].

This article concerns the selection of the most appropriate model of the distribution of measurement points located on a free-form surface. The purpose of the new method of determining the location of measurement points on curvilinear surfaces is to improve the performance of contact coordinate measurements by reducing the time of measurements and increasing their accuracy. The improvement of measurement accuracy is related to determining the correct location of measurement points on measured surfaces of products. Measurement points should be arranged in the places of products characterized by the highest values of form deviations. The assumed distribution of measurement points should enable the measurement of real form deviations of a measured product.

The article presents the new method of the distribution of measurement points on curvilinear surfaces of products. The best distribution of measurement points was selected by using the Analytic Hierarchy Process (AHP), which has already been applied in the coordinate measuring technique when e.g., selecting a coordinate measuring machine [7]. The results of simulation investigations, which are presented in the work [8] also indicate that the AHP can be used when planning the strategy of contact coordinate measurements. The AHP allows decomposing a complex decision problem, regarding e.g., selecting locations of measurement points on measured surfaces, and creating a final ranking for a finite set of considered variants. In addition, the AHP analysis has also been successfully used in many other areas, not just only for measurements.

The developed method takes into account various aspects of coordinate measurements of products built of curvilinear surfaces, which makes it very universal, and thus it can be used in the case of measurements of products characterized by different geometrical shapes produced by companies from different industries. Curvilinear surfaces are used e.g., in the aviation and automotive industries [1]. The quality of free-form surfaces of products is constantly increasing by e.g., improving their machining processes [9]. Free-form surfaces can be measured by using different coordinate measuring systems, e.g., CMMs or CNC machine tools equipped with non-contact measuring probes [10]. Measurements carried out by using coordinate measuring machines are characterized by the highest measurement accuracy and the results obtained by using these systems can be treated as reference ones when analyzing the results of measurements carried out by using other measuring systems [10].

The created method is the extension of the approach to selecting measurement points shown in the work [8], which presents only the results of simulation research. Moreover, the new method takes into account the new criteria for selecting the way of distributing measurement points on free-form surfaces of products. In addition, the developed method, in contrast to the results presented in the article [8], was experimentally verified during real coordinate measurements of a product containing a curvilinear surface.

The selection of the methods of the distribution of measurement points located on curvilinear surfaces is still a current topic in the coordinate metrology. There are many algorithms of determining the distribution of measurement points in the case of measurements conducted by using coordinate measuring systems working in the contact mode. In general, there are two main ways of distributing points. The first one is called the uniform distribution. In the case of the second method, measurement points are distributed non-uniformly. The following paragraphs of this section present the examples of methods of the determination of locations of measurement points on surfaces of measured objects.

The method of the distribution of measurement points on curvilinear surfaces of products is presented e.g., in the work by Mingrang et al. [11]. The algorithm of localization of points is based on the form error model, which was created by adding deviations to a nominal model of an investigated product. The proposed method was compared to two often applied methods of selecting locations of measurement points on measured free-form surfaces: the uniform distribution of points and the method based on the curvature of a product. The presented method gained the best results concerning the applied number of measurement points and the time of measurements.

Three methods of the distribution of points were analyzed by Lalehpour et al. [12]. The points were distributed on selected surfaces. One of the considered methods was the random distribution of measurement points. The investigations were performed for different numbers of points and by using the virtual sampling. The analyses were conducted to select the best method of the distribution of measurement points. The best method includes the smallest number of measurement points which accurately represent an investigated surface.

ElKott et al. [13] presented four methods of distributing measurement points, which are based on, e.g., the uniform distribution, the areas of surface patches and the curvature of a patch.

The paper by ElKott and Veldhuis [14] and the work by ElKott [15] present two different methods of positioning measurement points on surfaces measured by a CMM. The developed methods are as follows: the method based on deviations calculated between a nominal model and a substitute model and the method taking into account the curvature of a considered surface.

New strategies of finding the locations of measurement points were also proposed by Rajamohan et al. [16,17]. They are based on the lengths of investigated curves and the areas of measured surfaces. Moreover, the authors applied dominant points, at which the maximum local curvatures occur.

The results of investigations regarding the selection of measurement points in the case of coordinate measurements of a blade were presented by Jiang et al. [18]. Three methods of the distribution of points were compared and the chordal deviation method obtained the best results.

The literature concerning the coordinate measuring technique lacks automated methods for selecting the location of measurement points on measured surfaces of products that could be directly implemented in commercial metrological software cooperating with, e.g., coordinate measuring machines equipped with contact measuring probes. The developed method of defining the distribution of measurement points, which is shown in this paper, can be easily implemented in commercial measurement software. This implementation contributes to increasing the chances of using the new method under industrial conditions.

The following sections of the paper concern, e.g., the presentation of the proposed method of the distribution of measurement points, the applied structure of the analytic hierarchy process, the explanation of considered criteria used during the AHP analysis, the results of performed research and the conclusions regarding the selection of the best model of the distribution of measurement points.

## 2. Proposed Method of the Distribution of Measurement Points

**Figure 1:**
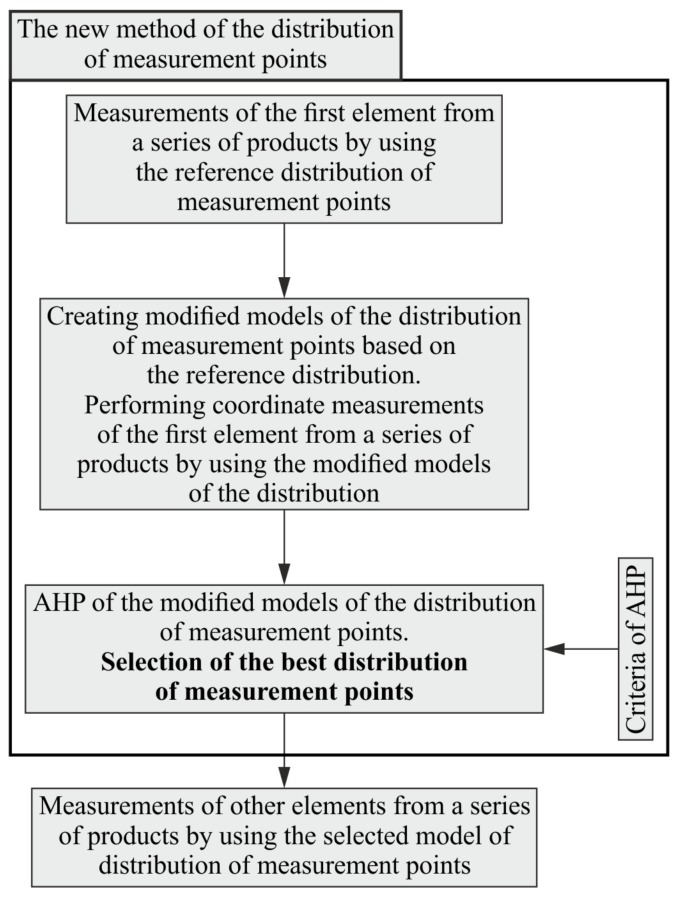
The stages of measurements of a series of products when using the new method of determining the location of measurement points.

The proposed method of determining the location of measurement points on free-form surfaces of measured objects is dedicated primarily to coordinate measurements of a series of manufactured products. Its fundamental advantage, which is associated with shortening the time of coordinate measurements, is visible when it is necessary to carry out measurements of the whole series of machined products not differing in terms of their nominal shapes. The length of shortened time results from the number of measured objects. The new method requires the use of the so-called reference model of the distribution of measurement points, which should be used in the case of coordinate measurements of the first object being a part of a series of manufactured products. The created method of the distribution of points consists of three stages. They are presented in the first part of Figure 1. Moreover, Figure 1 presents the steps of coordinate measurements of a series of investigated products when applying the developed method.

The initial stage of the proposed method for defining the location of measurement points is the coordinate measurement of the first workpiece from a given series of products by using the mentioned reference distribution of measurement points. The reference distribution should include the largest possible number of measurement points, which may lead to the highest possible measurement accuracy. A large number of measurement points increases the chances of identifying real form deviations of a measured object.

In the next stage, the so-called modified distributions of measurement points, including the smaller number of points compared to the reference distribution, are created. They are formed based on the reference distribution of measurement points. A smaller number of measurement points, especially in the case of contact coordinate measurements, leads to a reduction in the time of measurements, which may contribute to increasing the efficiency of the entire manufacturing process of a given product. Modified distributions can be created in a random manner by random elimination of measurement points included in the so-called reference distribution. In the second stage, the first object from a given series of manufactured products should also be measured by using the analyzed modified distributions of measurement points.

The third stage of the created method of defining the distribution of points concerns performing the AHP analysis based on the adopted criteria. The purpose of implementing the multi-criteria analysis is to choose the best distribution of measurement points from the group of analyzed and modified, in relation to the reference distribution, models of the location of measurement points lying on a measured free-form surface of a product.

After conducting the AHP analysis, the rest of objects from analyzed series of products should be measured by means of a selected modified distribution of measurement points, as shown in the second part of Figure 1. The modified distribution includes a smaller number of points compared to the reference distribution. This, in turn, reduces the time of contact coordinate measurements of curvilinear surfaces of products. Speeding up a measurement task by using a smaller number of measurement points is mainly visible when measurements are carried out in the single-point probing mode. This mode of coordinate measurements is still widely used in the industrial practice, e.g., during measurements carried out with the use of coordinate measuring machines or CNC machine tools.

## 3. AHP Hierarchical Structure

The most important stage of the proposed new method of defining the distribution of measurement points on measured curvilinear surfaces of objects is the third one, which assumes the application of the multi-criteria analysis of the considered modified models of the distribution of measurement points located on measured free-form surfaces.

The goal of the AHP analysis was the selection of the best model of the distribution of measurement points lying on a free-form surface. To select the most appropriate distribution of measurement points the following criteria were used:

- form deviations calculated by using the applied model of the distribution of measurement points,
- number of measurement points belonging to the considered model of the distribution,
- accuracy of substitute models created based on the measurement points located on a free-form surface,
- time of contact coordinate measurements,
- areas of 3D substitute models representing the models of the distribution of measurement points.

**Figure 2:**
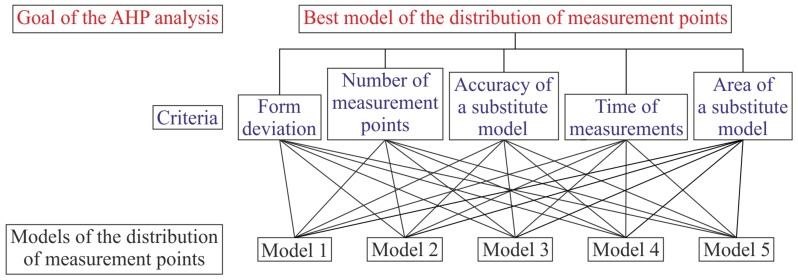
AHP hierarchical structure applied to select the best model of the distribution of measurement points.

The general view of the AHP hierarchical structure, which was used during performed investigations for the multi-criteria prioritization process in order to select the best model of the distribution of measurement points located on a free-form surface of a measured object, is presented in Figure 2.

The first criterion used during the AHP analysis is associated with the form deviations calculated by using the considered models of the distribution of measurement points located on a free-form surface. The form deviations obtained by means of the analyzed models of the distribution were compared to the reference form deviation measured with the use of a coordinate measuring machine and the reference distribution of points which includes the largest number of uniformly distributed measurement points. The first considered criterion helps the user of a coordinate measuring system to assess whether the applied distribution of measurement points can detect the highest values of form deviations of measured curvilinear surfaces of objects. In the case of the first criterion, the form deviation measured by using the best model of the distribution should be as close as possible to the reference deviation.

The second criterion applied during the prioritization process of the models of the distribution of measurement points is related to the time of coordinate measurements performed by using CMMs. The time of measurements in the case of the single-point probing mode depends on the numbers of measurement points and scanning lines, along which measurement points are located, used to measure a free-form surface. The time of coordinate measurements is one of the factors with the influence on the efficiency of measurements. The users of coordinate measuring machines must decide how to conduct measurements to obtain the highest possible accuracy of coordinate measurements in relatively short time. The time of measurements is the part of the entire time of the production process of a product. Therefore, for the second criterion, the most favorable distribution of points is provided by the model for which the measurement time is the shortest.

The next applied criterion is the number of measurement points located on a curvilinear surface of a measured object. The accuracy of contact coordinate measurements is directly connected with the amount of data representing a measured product. A large number of measurement points increases the probability of measuring the highest of existing form deviations characterizing the quality of a measured product. Therefore, the model with the largest number of measurement points is the best one. However, if coordinate measurements are conducted in the single-point probing mode, the number of measurement point cannot be too large in order not to increase the time of coordinate measurements too much.

The fourth applied criterion is related to the accuracy of a substitute model of a measured free-form surface. The substitute models are fitted to the groups of measurement points being the parts of the considered models of the distribution of measurement points. The accuracy of the substitute models must be analyzed to check how the considered models of the distribution represent a measured free-form surface taking into consideration the geometrical complexity of curvilinear surfaces. Moreover, substitute models of measured curvilinear surfaces were successfully used by many researchers [13,14,15] in order to distribute measurement points on measured surfaces of an object, which makes such an approach to planning the strategy of contact coordinate measurements very popular in the coordinate measuring technique. In the case of the proposed AHP analysis and the fourth criterion, the best model of the distribution is the one which generates a substitute surface characterized by the highest accuracy.

The last of the analyzed criteria is the area of the substitute model representing measurement points distributed on an analyzed curvilinear surface. In the coordinate measuring technique, it is always better to spread measurement points on the largest possible fragments of measured free-form surfaces of an investigated object. This may increase the chances of detecting the highest form deviations of free-form surfaces, which are planned to be measured by using CMMs. For the last criterion, the model of the distribution of measurement points located on the largest possible area of a measured surface is the best one.

In the case of the proposed method of defining the distribution of measurement points on free-form surfaces of measured products, the selection of the best model of the distribution is carried out on the basis of the above-mentioned criteria of the AHP analysis. The models of the distribution assume different numbers and positions of points located on measured curvilinear surfaces. To present the possibilities of the method of determining the distribution of measurement points, both simulation and experimental investigations were carried out. Five different models of the distribution were taken into account when conducting the research. They are presented in detail in Section 4.2.

## 4. Simulation and Experimental Investigations

The conducted numerical and experimental investigations concern three stages of the created method of defining the distribution of measurement points on free-form surfaces of measured products. The analyzed stages are aimed at selecting the best distribution of measurement points among the considered models of the distribution. The best distribution of measurement points should ensure fast and accurate coordinate measurements of analyzed products. The purpose of the research was to verify the possibility of performing coordinate measurements of a series of manufactured products by using a measurement strategy, which includes a smaller number of points compared to the reference distribution of measurement points.

### 4.1. Analyzed Curvilinear Surface

**Figure 3:**
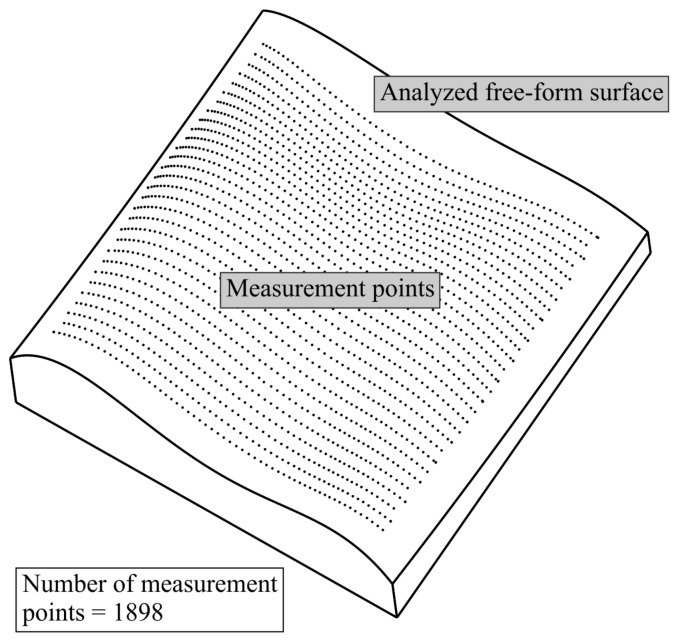
The view of the investigated free-form surface for which the best distribution of measurement points was searched and the reference model of the distribution of measurement points.

The investigations were carried out for the selected free-form surface shown in Figure 3. Its 3D model was created by using the CATIA V5-6 software and then imported into the Calypso metrology software of the Carl Zeiss company. In the Calypso software, the reference distribution of measurement points was declared. It includes the largest number of points, and thus it provides the highest probability of measuring the actual value of a form deviation of an analyzed curvilinear surface. The real object containing the analyzed free-form surface was made of aluminum alloy by using the DMU 100 monoBLOCK CNC machine tool. The dimensions of the bottom surface of the machined product were around 125×127 mm. The considered free-form surface of the product was the theoretical surface that was used to present the possibilities of the proposed method for defining the distribution of measurement points. Therefore, no technical documentation, including, e.g., geometrical tolerances, was available for this investigated surface. The values of tolerances were not needed for the presentation of the proposed method. However, the documentation of the machined product is, of course, necessary, but after applying the developed method and selecting measurement points, when the accuracy of the product should be assessed.

### 4.2. The Considered Models of the Distribution of Measurement Points

**Figure 4:**
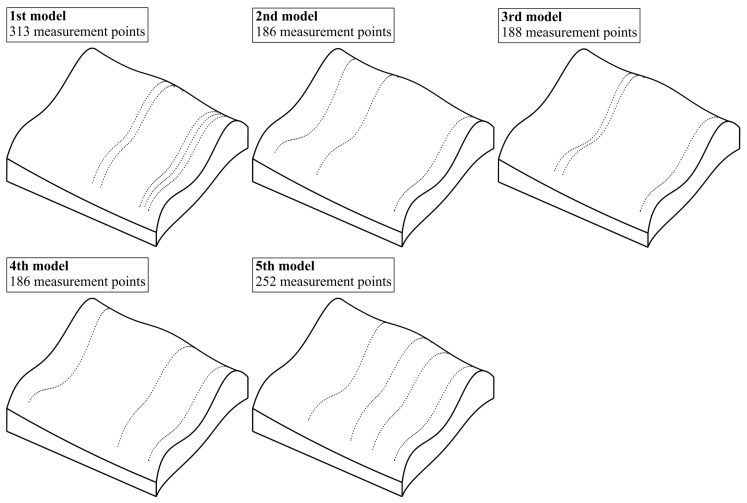
The analyzed models of the distribution of measurement points.

The best distribution of measurement points located on the selected free-form surface was chosen from the group of five different models of the distribution of measurement points. The models were randomly selected by an operator of a coordinate measuring machine. Figure 4 illustrates the considered models of the distribution. The models, similarly to the reference distribution of measurement points, were created by using the Calypso inspection software. The models were prepared by modifying the reference distribution of points presented in Figure 3. The modifications involved changing the amount of the scanning lines containing measurement points and distributed on the investigated free-form surface. The use of the models based on measurement points arranged along selected curves representing the analyzed curvilinear surface results from a scanning measuring probe applied during experimental research. However, the proposed method of determining the location of measurement points can also be used for other distributions of points (not only lying on selected curves) measured by using, e.g., the single-point probing mode.

Moreover, Figure 4 presents the number of measurement points included in the considered models of the distribution of measurement points. The final number of models of the distribution of measurement points considered in the proposed method must be selected by the user of a coordinate measuring system.

### 4.3. Results of Simulation Investigations

**Figure 5:**
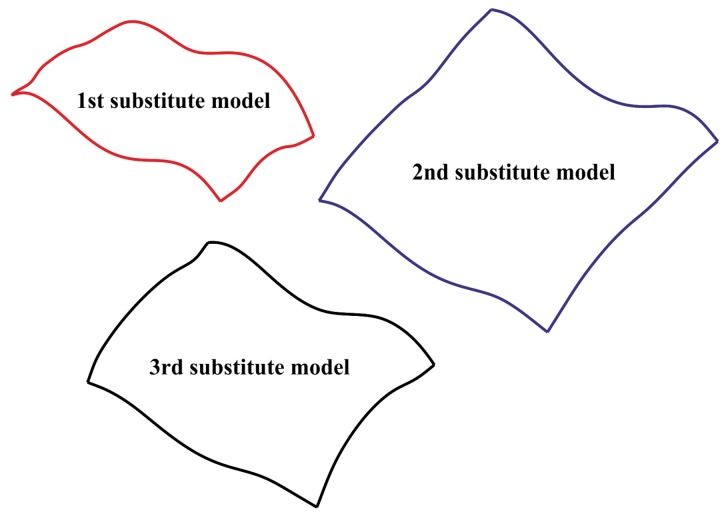
The selected substitute models representing the measured free-form surface.

The performed numerical research concerned two criteria of the AHP analysis. The first one was related to the determination of the accuracy of 3D substitute surfaces created for the considered curvilinear surface and the analyzed models of the distribution of measurement points. The second one was associated with the calculation of the areas of generated substitute surfaces. The substitute models of the considered free-form surface were created by using the Zeiss Reverse Engineering software. Moreover, they were prepared by means of the third-degree B-Spline surfaces fitted to measurement points being parts of the considered models of the distribution of points. Figure 5 presents the examples of the substitute surfaces, created based on the considered models of the distribution of measurement points, of the analyzed curvilinear surface. The boundaries of the presented substitute surfaces do not correspond to the boundaries of the nominal curvilinear surface due to the fact that the substitute models were created based on the different groups of measurement points, which were differently shifted from the boundaries of the analyzed free-form surface.

After creating the substitute models of the analyzed free-form surface, the next step was to calculate their areas, which was done by using the appropriate functions of the CATIA V5-6 software. The calculated surface areas for the individual substitute models, created by means of the mentioned Zeiss Reverse Engineering software, are presented in Table 1. The numbers of the substitute models, presented in Table 1, correspond to the numbers of the individual models of the distribution of measurement points. The need to calculate the surface areas of the substitute models was the result of using the last of the five criteria of the AHP analysis.

In the next step, the maximum deviations between the substitute models of the analyzed curvilinear surface and the geometrical model of the surface which was created on the basis of the reference model of the distribution of measurement points containing the largest amount of measurement data were calculated. The relative values of deviations calculated based on the maximum form deviations of the individual substitute models are presented in Table 2. The values presented in Table 2 were calculated by dividing the deviations of the models listed in the first row of the mentioned table by the deviations of the models, which are listed in the first column.

The results of the numerical investigations were necessary for conducting the third stage of the proposed method of defining the distribution of measurement points, i.e., to perform the AHP analysis aimed at selecting the best distribution of points on the measured curvilinear surface.

### 4.4. Results of Experimental Research

**Figure 6:**
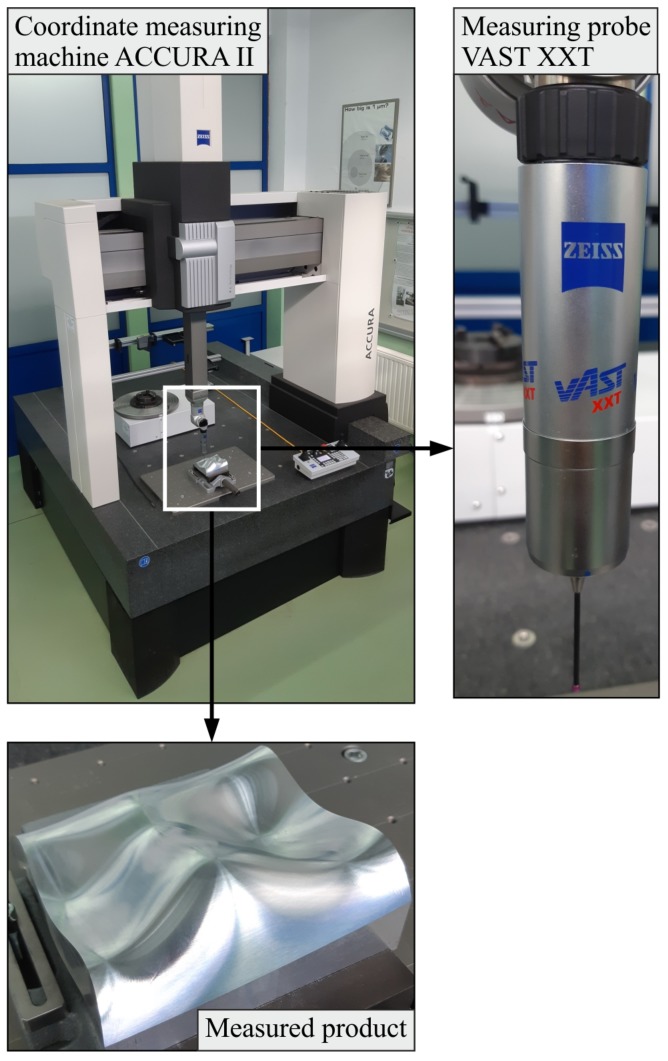
The applied coordinate measuring machine and the free-form surface measured by the CMM.

To conduct the AHP analysis regarding the considered models of the distribution of measurement points the experimental investigations were also conducted. During the experimental research, the values of form deviations and the time of coordinate measurements were registered. The values of the mentioned parameters, similarly to the results of numerical research, were necessary to perform the AHP analysis. The experimental investigations were conducted by using the ACCURA II coordinate measuring machine (Carl Zeiss, Oberkochen, Germany) equipped with the VAST XXT measuring probe (Carl Zeiss, Oberkochen, Germany) and the Calypso software. In the first stage of coordinate measurements the reference form deviation was measured by using the reference distribution of measurement points presented in Figure 3. In the next step of the experimental investigations, the subsequent form deviations were obtained by means of the considered five models of the distribution of measurement points. All form deviations were calculated by means of the appropriate functions of the Calypso inspection software. Moreover, the time of coordinate measurements of the mentioned deviations was registered. Figure 6 presents the coordinate measuring system applied during the experimental research and the investigated free-form surface.

The accuracy parameters of the applied coordinate measuring machine are as follows:

- $E_{L,MPE}=\left(1.6+L/333\right)$μm;
- $P_{FTU,MPE}=1.7$μm;
- $MPE_{Tij}=2.5$μm;
- $MPT_{\tau ij}=50.0$ s;

where: $E_{L,MPE}$—a maximum permissible error of a length measurement; *L*—a measured length, mm; $P_{FTU,MPE}$—maximum permissible single-stylus form error; $MPE_{Tij}$—a maximum permissible scanning error; $MPT_{\tau ij}$—maximum permissible scanning test duration.

The analysis of the results of coordinate measurements was carried out without the best-fit of the measurement results to the nominal data. The measurements were carried out in the scanning mode with the scanning speed of 10 mm/s, the distance between measurement points was 2 mm. The results of experimental studies, in the form of the measured deviations and the times of individual measurements, are presented in Table 3. The time of coordinate measurement carried out by using the reference distribution of measurement points was equal to 514 s.

Large deviations from the reference model, presented in the second column of Table 3 and calculated for the first, third and fifth models of the distribution of measurement points, are the results of the location of points in the places of the analyzed curvilinear surface where the largest form deviations do not occur. The form deviations obtained during coordinate measurements conducted by using the second and fourth models were very close to the deviation measured on the basis of the reference model of the distribution of measurement points because the points were in the areas characterized by the worst quality. The value of the reference deviation was equal to 0.1955 mm. In the case of the reference model, there is the highest probability of identifying the worst-made fragments of the measured free-form surface due to a large number of measurement points.

The results of experimental research, similarly to the results of the simulation investigations, were used to select the best (i.e., accurate and fast) measurement strategy.

## 5. AHP Analysis of the Considered Models of the Distribution

The AHP analysis was performed to select the most appropriate model of the distribution of points when conducting coordinate measurements of the selected free-form surface. At the beginning, the comparisons of the criteria in relation to the goal (i.e., the selection of the optimal distribution of measurement points) by using the nine-point scale were conducted. The pairwise comparisons were carried out by using the scale presented in Table 4 [19]. The results of the comparisons are presented in Table 5 and they derived from the experience of the user of a coordinate measuring system.

The consolidated priorities regarding the considered criteria are presented in Table 6. It has been revealed that comparisons do not demonstrate inconsistency, as the consistency ratio (CR) is 8.9%.

In the next step, the comparisons of the alternatives with respect to each criterion were made. To compare the alternatives the normalization of the data calculated by using the CATIA V5-6 and Calypso software packages to the range from 1 to 9 was applied. Table 7, Table 8, Table 9, Table 10 and Table 11 present the results of the comparisons of the models of the distribution. The comparisons, similarly to the comparison of the criteria, do not demonstrate inconsistency. The largest value of the CR parameter was identified for the area criterion and it was equal to 2.1%.

**Figure 7:**
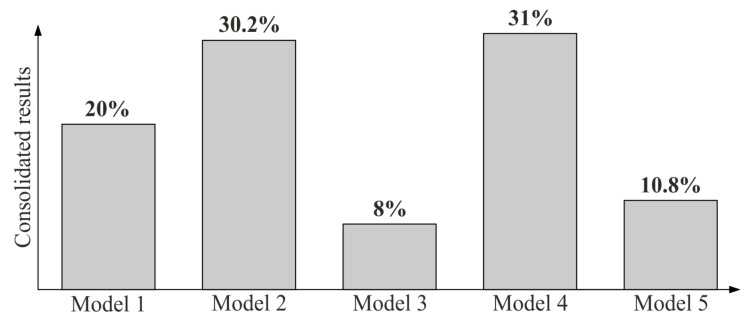
The consolidated weights of the analyzed models of the distribution of measurement points.

Table 12 presents the sequence of the alternatives (i.e., the considered models of the distribution of measurement points), based on the weights calculated with respect to each criterion, after performing the AHP analysis. Moreover, Figure 7 presents the consolidated weights of the alternatives that have been derived by taking the criteria and alternatives-based pairwise comparisons into consideration.

Table 5, Table 6, Table 7, Table 8, Table 9, Table 10, Table 11 and Table 12 are the result of the sequence of actions that should be carried out when using the Analytic Hierarchy Process in order to choose the best distribution of measurement points.

Based on the AHP analysis, the best distribution of measurement points on the considered curvilinear surface, taking into account all analyzed criteria, is represented by the fourth model. Models 1–3 and model 5 have subsequent priorities. Hence, model 4 shall be used in practical coordinate measurements of a series of manufactured products. It is also allowed to use the second model of the distribution of measurement points, which achieved the very similar result to the fourth model.

## 6. The Time of Coordinate Measurements Carried Out by Using the New Method of the Distribution of Measurement Points

The significant profit resulting from the application of the proposed method of defining the distribution of measurement points on free-form surfaces of products is immediately visible for even a small series of measured products (e.g., composed of ten products). In the case of the created method, the first object from a given series of products should be measured by using a reference distribution of measurement points (i.e., the first stage of the method) and with the use of models of the distribution of measurement points generated by modifying a reference distribution (i.e., the second stage of the method). The measurement conducted by using a reference distribution should identify the existing true value of a deviation of a measured product.

Assuming the measurement of the surface, the shape of which is presented in Section 4.1 and the analysis of five measurement point distribution models presented in Section 4.2, the total measurement time of the first product from the series of ten measured products would be 885 s by using the new method of determining the distribution of measurement points. The calculated time results from the sum of the times of coordinate measurements of the first product performed by using the reference distribution and the five considered models of the distribution of measurement points.

Based on the AHP analysis, which is implemented in the third stage of the proposed method of defining the location of points, the fourth model of the distribution of measurement points should be used in the case of real coordinate measurements of the next nine objects included in the considered series composed of ten products. The application of the fourth model of the distribution of measurement points generates the measurement time for the considered free-form surface of a single product equal to 61 s. Therefore, for the series of ten products, the total time of their coordinate measurements would be 1434 s assuming the application of the proposed method of defining the distribution of measurement points located on surfaces of measured objects. This time is the result of the sum of the total measurement time of the first product (i.e., 885 s) and the sum of the measurement times of the other nine items that are the part of the given series of products and which should be measured by means of the fourth measurement point distribution model (i.e., 549 s).

**Figure 8:**
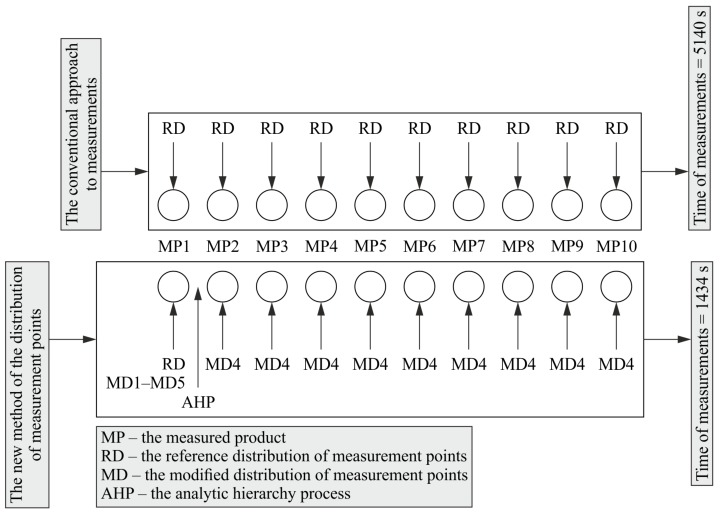
The benefit of using the new method of the distribution of measurement points.

In the case of not using the new method for determining the location of measurement points, all ten items of the given series of products should be measured by using the reference distribution of measurement points, which would generate the total measurement time of all ten products equal to 5140 s. This time is almost four times longer than the total measurement time of coordinate measurements of ten workpieces when using the new method of defining the distribution of measurement points on curvilinear surfaces. The graphical representation of the benefit of using the new method of the distribution is shown in Figure 8. The figure presents the comparison of the time of measurements when using the developed method with the time of measurements of all products conducted by means of the reference distribution.

## 7. Conclusions

The article presents the new method for determining the location of measurement points on free-form surfaces of measured products. The results of the performed investigations and the analysis carried out by using the AHP method indicate the possibility of a significant reduction in time of coordinate measurements of a series of products when using the new method for determining the distribution of measurement points located on measured free-form surfaces. The time reduction is the result of decreasing the number of measurement points for most products that are the part of a given series of workpieces. Moreover, a smaller number of measurement points does not reduce the accuracy of coordinate measurements, which results from taking into account various criteria of the considered AHP analysis, e.g., ‘the time of coordinate measurements’ and ‘the form deviation of a measured surface’, when determining the location of measurement points by using the proposed method.

The advantages of the proposed method of the distribution of measurement points on curvilinear surfaces are the increase in the level of automation of defining the position of measurement points and the possibility of implementing the developed method in commercial measurement software, so that it can be used in industry. The example of popular measurement software in which the new method can be relatively easily applied is the Calypso software package. The application of the created method of determining the location of measurement points in the Calypso inspection software is possible by means of its module of parametric programming of coordinate measurements—PCM (parameter coded measurements).

The further research should be aimed at testing the proposed method of determining the location of measurement points under real industrial conditions and the implementation of the proposed method in commercial measurement software.

## Figures and Tables

**Table 1:** The surface areas of the substitute models.

Substitute Model	Surface Area, ${\mathrm{mm}}^{2}$
Substitute model 1	7046
Substitute model 2	13,935
Substitute model 3	10,364
Substitute model 4	14,292
Substitute model 5	10,624

**Table 2:** The relative values of the form deviations of the substitute models.

	Model 1	Model 2	Model 3	Model 4	Model 5
Model 1	1	2.3	3.7	2.7	1.7
Model 2	0.4	1	1.6	1.1	0.7
Model 3	0.3	0.6	1	0.7	0.5
Model 4	0.4	0.9	1.4	1	0.6
Model 5	0.6	1.4	2.2	1.6	1

**Table 3:** The results of experimental investigations.

Model	Deviation from the Reference Model, mm	Time, s
Model 1	0.0570	99
Model 2	0.0058	63
Model 3	0.0721	66
Model 4	0.0056	61
Model 5	0.0653	82

**Table 4:** The scale for performing pairwise comparison [19].

Intensity of Importance	Definition	Explanation
1	Equal importance	Two factors (A and B) are equally important
3	Moderate importance	Experience and judgment favor A over B
5	Strong importance	A is strongly favored over B
7	Very strong importance	A is very strongly favored over B
9	Extreme importance	The evidence favoring A over B is of the highest possible validity
2, 4, 6, 8	Intermediate values between judgments	A compromise is required

**Table 5:** The comparisons of the criteria.

	Form Deviation	Time	Number of Points	Substitute Model	Area
Form deviation	1	5	6	5	5
Time	0.2	1	3	0.33	3
Number of points	0.17	0.33	1	0.25	2
Substitute model	0.2	3	4	1	4
Area	0.2	0.33	0.5	0.25	1

**Table 6:** The consolidated priorities concerning the criteria.

Category	Priority	Rank
Form deviation	53.9%	1
Time	12.3%	3
Number of points	6.7%	4
Substitute model	21.8%	2
Area	5.4%	5

**Table 7:** The comparisons of the alternatives with respect to a form deviation.

	Model 1	Model 2	Model 3	Model 4	Model 5
Model 1	1	0.14	1	0.14	1
Model 2	7	1	9	1	8
Model 3	1	0.11	1	0.11	1
Model 4	7	1	9	1	8
Model 5	1	0.12	1	0.12	1

**Table 8:** The comparisons of the alternatives with respect to time.

	Model 1	Model 2	Model 3	Model 4	Model 5
Model 1	1	0.12	0.14	0.11	0.33
Model 2	8	1	1	1	5
Model 3	7	1	1	0.5	4
Model 4	9	1	2	1	5
Model 5	3	0.2	0.25	0.2	1

**Table 9:** The comparisons of the alternatives with respect to a number of points.

	Model 1	Model 2	Model 3	Model 4	Model 5
Model 1	1	9	9	9	4
Model 2	0.11	1	1	1	0.2
Model 3	0.11	1	1	1	0.2
Model 4	0.11	1	1	1	0.2
Model 5	0.25	5	5	5	1

**Table 10:** The comparisons of the alternatives with respect to an area.

	Model 1	Model 2	Model 3	Model 4	Model 5
Model 1	1	0.11	0.2	0.11	0.2
Model 2	9	1	4	1	3
Model 3	5	0.25	1	0.25	1
Model 4	9	1	4	1	4
Model 5	5	0.33	1	0.25	1

**Table 11:** The comparisons of the alternatives with respect to a substitute model.

	Model 1	Model 2	Model 3	Model 4	Model 5
Model 1	1	5	9	6	3
Model 2	0.2	1	2	1	0.5
Model 3	0.11	0.5	1	0.5	0.25
Model 4	0.17	1	2	1	0.5
Model 5	0.33	2	4	2	1

**Table 12:** The hierarchy with the consolidated priorities.

	Model 1	Model 2	Model 3	Model 4	Model 5
Form deviation	0.056	0.421	0.050	0.421	0.053
Time	0.033	0.299	0.243	0.353	0.071
Number of points	0.597	0.055	0.055	0.055	0.238
Substitute model	0.545	0.103	0.053	0.100	0.199
Area	0.031	0.358	0.112	0.381	0.118
